# Production and quality control ^177^Lu (NCA)—DOTMP as a potential agent for bone pain palliation

**DOI:** 10.1120/jacmp.v17i6.6375

**Published:** 2016-11-08

**Authors:** Nafise Salek, Mojtaba Shamsaei, Mohammad Ghannadi Maragheh, Simindokht Shirvani Arani, Ali Bahrami Samani

**Affiliations:** ^1^ Faculty of Energy Engineering and Physics Amirkabir University of Technology Tehran Iran; ^2^ Nuclear Fuel Cycle Research School Nuclear Science and Technology Research Institute(NSTRI) Tehran Iran

**Keywords:** ^177^Lu, no‐carrier‐added, DOTMP, radiopharmaceutical, biodistribution

## Abstract

Skeletal uptake of radiolabeled‐1, 4, 7, 10‐tetraazacyclododecane‐1, 4, 7, 10‐tetramethylene phosphoric acid (e.g., ^177^Lu‐DOTMP) complex, is used for bone pain palliation. The moderate energy of β‐emitting ${\;}^{177}\text{Lu}\;(T_{1/2}=6.7\;d,E_{\beta \text{max}}=497\;\text{keV})$ has been considered as a potential radionuclide for development of the bone‐seeking radiopharmaceutical. Since the specific activity of the radiolabeled carrier molecules should be high, the “no‐carrier‐added radionuclides” have significant roles in nuclear medicine. Many researchers illustrated no‐carrier‐added ^177^Lu production; among these separation techniques such as ion exchange chromatography, reversed phase ion‐pair, and electrochemical method, extraction chromatography has been considered more capable than other methods. In order to optimize the conditions, some effective factors on separation of Lu/Yb were investigated by EXC. The NCA ^177^Lu, produced by this method, was mixed with 300μ1 of DOTMP solution (20 mg in 1 mL of 0.5 M NaHCO_3_, pH=8) and incubated under stirring at room temperature for 45 min. Radiochemical purity of the ^177^Lu‐DOTMP complex was determined using radio‐thin‐layer chromatography (RTLC) method. The complex was injected to wild‐type rats and biodistribution was then studied for seven days. The NCA ^177^Lu was produced with specific activity of 48 Ci/mg and with a radinuclidic purity of 99.99% through irradiation of enriched ^176^Yb target (1 mg) in a thermal neutron flux of $4\times 10^{13}\;n.{\text{cm}}^{-2}.s^{-1}$ for 14 days. ^177^Lu‐DOTMP was obtained with high radiochemical purities (>98%) under optimized reaction conditions. The radiolabeled complex exhibited excellent stability at room temperature. Biodistribution of the radiolabeled complex studies in rats showed favorable selective skeletal uptake with rapid clearance from blood along with insignificant accumulation within the other nontargeted organs.

PACS number(s): 87.57.un, 87.57.uq

## I. INTRODUCTION

Cancer cells often metastasize from their original site (such as the breast or prostate cancers) to the bones. Many cancer patients will suffer from bone metastases which are accompanied by pain, bone fractures, spinal cord compression, hypercalcemia, and rapid degradation in quality of life.^1^, ^2^, ^3^ Standard methods to treat bone metastases include systemic therapies (the use of analgesics and bisphosphonates, chemotherapy, and hormonal therapy) and local control (radiation therapy using an external beam), and radiofrequency ablation along with the surgical stabilization of the affected sites.^(4)^ The use of suitable radionuclides linked to bone‐specific ligands has an important role in palliating pain of bone metastases due to the numerous limitations of the other therapeutic methods.^(5)^ It is critically important with effective palliative bone‐targeted radiopharmaceuticals to ensure their selective uptake at the skeletal lesion sites while keeping the absorbed doses by the bone marrow as low as possible.^(6)^ The two most important criteria that determine the utility of any bone‐targeted radiopharmaceutical in a given situation are which radionuclide is being used and which site‐specific carrier is included.^(7)^ Phosphonates carriers, such as EDTMP(diethylenetriamine penta(methylene phosphonic acid)), DOTMP(1,4,7,10‐tetraazacyclododecane‐1,4,7,10‐tetramethylene phosphonic acid), APD(1‐hydroxy‐3‐ amino propylidene‐diphosphonic acid), TTHMP(Triehtylenetetramine hexamethylene phosphonate), are being used for the other radiopharmaceuticals that are site‐specific for skeletal lesions.^6^, ^7^, ^8^, ^9^, ^10^, ^11^, ^12^, ^13^, ^14^, ^15^, ^16^, ^17^ Low‐energy β^‐^emitting radionuclides, such as ^177^Lu, ^153^Sm, ^175^Yb, and ^186^Re, are used for palliation of bone pain, whereas radionuclides with higher energies including ^166^Ho, ^90^Y, and ^188^Re are recommended for bone marrow ablation. Sometimes, the carrier and radionuclide are one and the same (as ^32^P and ^89^Sr) because of their similarity to the elemental composition of bone.^17^, ^18^, ^19^, ^20^, ^21^

Since 1,4,7,10‐tetraazacyclododecane‐1,4,7,10‐tetramethylene phosphonic acid (DOTMP) has more thermodynamic stability and forms kinetically inert complexes with lanthanides compared to its acyclic analogs, it is selected as the ligand.^(10)^

^177^Lu is suitable for palliation of bone pain due to its excellent radionuclide properties. ^177^Lu decays with a half‐life of 6.71 days by emission of β‐particles with E_max_ of 497 keV (78.6%), 384 keV (9.1%), and 176 keV (12.2%) and ^177^Hf is formed. It also can emit gamma photons of 113 keV (6.4%) and 208 keV (11%), which are suited for nuclear imaging for the purpose of *in vivo* localization.^(22)^ The significant advantage of utilizing ^177^Lu is the energies of its β‐particles, which are adequately low; it is expected to have minimum bone‐marrow suppression after accumulation in skeletal lesions.^23^, ^24^ The optimal half‐life of ^177^Lu makes it as a useful tool for long‐distance shipping and also provides enough time to produce the ^177^Lu‐based radiopharmaceuticals.^(25)^

Usually, two alternative production routes are applied to obtain ^177^Lu: namely, the direct route is based on the neutron irradiation of lutetium targets, and the indirect route is based on the neutron irradiation of ytterbium targets followed by radiochemical separation of ^177^Lu from ytterbium isotopes.^(26)^ Formation of a small amounts of long‐lived ^177^mLu $\left(t_{1/2}=160.5\;d\right)$ is the main drawback of the direct route. Using this method, the product will also contain macro quantities of nonradioactive isotopes of Lu and, consequently, has a comparatively low specific activity.^27^, ^28^ With the indirect route, it is feasible to separate ^177^Lu from ^176^Yb due to their chemical differences, which leads to produce a “no‐carrier‐added” (NCA) therapeutic radioisotope of ^177^Lu without any nonradioactive isotope. For these reasons, the indirect process is preferred to produce Lu using ^176^Yb.

Many researchers reported separation of NCA ^177^Lu from Yb target by different methods.^26^, ^27^, ^28^, ^29^, ^30^, ^31^, ^32^, ^33^, ^34^, ^35^, ^36^, ^37^, ^38^, ^39^, ^40^, ^41^, ^42^, ^43^, ^44^, ^45^, ^46^, ^47^, ^48^, ^49^ In this study, NCA ^177^Lu is separated from ^176^Yb target by extraction chromatography (EXC). EXC is a conceptual flowsheet to separate the ^177^Lu/^176^Yb mixture based on the use of two different EXC resins; the resins contain either HEH (EHP) (LN2) or tetraoctyldiglycolamide (DGA) adsorbed on Amberchrom CG‐71 substrate. NCA 177Lu has been produced by EXC procedure and then its suitability for the preparation of radiochemical agents has been determined by preparing ^177^Lu‐DOTMP complexes as bone pain palliation agents.

## II. MATERIALS AND METHODS

### A. Materials and instruments

Isotopically enriched ^176^Yb_2_O_3_ (^176^Yb: 96.40%) was supplied by TRACE Sciences International (Richmond Hill, Ontario, Canada). LN2 resin (25–53 μm particle size) and DGA resin (50–100 μm particle size) were purchased from Eichrom Technologies Inc. (Lisle, IL), hydrochloric acid and nitric acid were obtained from Merck Company (Kenilworth, NJ). DOTMP and the other chemicals were obtained from Fluka Chemie GmbH (Buchs, Switzerland). Whatman No. 2 paper was used as chromatography papers. Radio‐thin‐layer chromatography (RTLC) was performed by the use of Whatman No. 2 papers using a thin‐layer chromatography scanner, Bioscan AR2000 (Bioscan Europe Ltd., France). All chemical reagents were of analytical grade. A p‐type coaxial HPGe detector (Eurasis Measure Company, NY City, NY), with 80% relative efficiency, a standard NIM, and resolution 1.8 keV at gamma ray energy 1332.5 keV of ^60^Co was used in this research. Length and diameter of the crystal were about 69 cm and 65 cm, respectively. The Gamma‐2000 software was also utilized for data acquisition and analysis, as well as MATLAB (MathWorks, Natick, MA) and Table Curve software, versions R2011b (7.13.0.564) and 5.01 (Systat Software Inc., San Jose, CA), respectively. Quantitative gamma counting was performed on an EG&G/ORTEC (Model 4001M, Jackson, MS) Mini Bin and Power Supply (NaI (Tl) counter. All values were expressed as mean ± standard deviation (Mean ± SD), and the data were compared using Student's *t*‐test. Finally, p‐values <0.05 were considered statistically significant. Animal studies were performed in accordance with the United Kingdom Biological Council's Guidelines.^(50)^ The animals were obtained from animal house of NSTRI, with mean age of nine ± one week and of the male gender.

### B. Irradiation

NCA ^177^Lu was produced through neutron irradiation of enriched ^176^Yb target in a quartz ampule with a thermal neutron flux of $4\times 10^{13}\;n.{\text{cm}}^{-2}.s^{-1}$ for 14 days at the Research Reactor of Tehran. ^175^Yb ($T_{1/2}=4.185$ days) was also produced due to the presence of ^174^Yb in the target and was used as a tracer for ytterbium. The irradiated target was cooled for two day to allow the decay of ^177^Yb ($T_{1/2}=1.9$ hrs). Then, the irradiated target was dissolved in HNO_3_ (0.1 N) for EXC separation.

### C. EXC separation

The system used for EXC separation had two glass columns (inner diameter of 11 mm and 22 cm bed height) that a layer of glass wool was inserted as the top bed support. The No. 1 glass column was thermostated at 50°C using recirculating water. A peristaltic pump and a connected polyethylene tube were used for passing solutions through the columns. To optimize the condition of this separation, LN2 resin (about 10 g with particles size of 25–53 μm) and DGA resin (10 g with the particles size of 50–100 μm) were wetted in dilute nitric acid (0.1 N) for 24 hrs. The both columns 1 and 2 with end capped glass wool were filled with well‐wetted LN2 and DGA resins, respectively. The columns were then preconditioned with distilled water (50 mL), HNO_3_ (50 mL, 0.1 N) for column 1 and HCl (50 mL, 0.05 N) for column 2 and again distilled water (50 mL), separately. The irradiated target in 0.1N HNO_3_ (15.4 mCi ^177^Lu and 2.7 mCi ^175^Yb) was loaded on the column 1 at a flow rate of 2 ml/min, was washed with 0.1N HNO_3_ and 1.5N HNO_3_, and was eluted with 4N HNO_3_. Column 2 was washed with 0.1N HNO_3_ and was eluted with 0.05N HCl. The eluted solution was collected in 5 mL bed volume and analyzed for Yb and Lu radionuclide using the HPGe detector.

#### C.1 The weight dependence of the Yb target

The effect of the initial mass of ytterbium loaded on the column was studied for the amount of 5 mg, 10 mg, and 20 mg. This different amount of Yb and 1 mg of Lu were introduced to separation system and ppm of Lu and Yb was checked to evaluate of effect of weight dependence of the Yb target on EXC separation.

#### C.2 The influence of the column temperature during EXC

The effect of two temperatures 30°C and 50°C was investigated on separation of Lu/Yb by using a circulator to adjusting the temperature.

#### C.3 Flow rate of load and elution

Rates of loading the target (1, 2, 5, and 7 mL/min) and eluting the system (2, 5, and 7 mL/min) were optimized on separation of Lu/Yb by adjusting the peristaltic pump.

### D. Radiolabeling of the DOTMP with NCA ^177^Lu

DOTMP solution was prepared by dissolving the ligand (20 mg) in NaHCO_3_ buffer (1 mL, 0.5 M, pH 8). NCA ^177^Lu (in 0/05N HCL) was obtained as the main product from the EXC separation system. NCA ^177^Lu (74 MBq) was then added to a conical vial and dried under a flow of nitrogen. The distilled water was added to the vial containing ^177^Lu and the activity followed by drying the vial using nitrogen flow (two times). Afterwards, the DOTMP solution (300 μL) was added to ^177^Lu vial. The pH of final solution was adjusted to 6–7. The reaction mixture was incubated under stirring at room temperature for 45 min. The radiolabeling efficiency experiments including radio‐thin‐layer chromatography, *in vitro* stability studies, and biodistribution studies were carried out to evaluate the complexing yield of ^177^Lu‐DOTMP over a period of time after production.

### E. Quality control of the product

#### E.1 Control of the radionuclide purity

Gamma ray spectroscopy was employed to measure the radionuclide purity of the final sample by an HPGe detector coupled to a Canberra multichannel analyzer (Canberra Industries Inc., Meriden, CT) for 1,000 sec.

#### E.2 Radio‐thin‐layer chromatography (RTLC)

A 5 μL sample of ^177^Lu‐DOTMP vial was spotted on the Whatman No. 2 chromatography paper as the stationary phase, and the saline solution was used as the mobile phase to discriminate free ^177^Lu from the radiolabeled compounds.^(11)^

#### *E.3* In vitro *stability studies*

The *in vitro* stability of the ^177^Lu‐DOTMP was studied by incubating the complex at room temperature in pH ~ 7 for a period time of 30 days (> four half‐lives of ^177^Lu) after preparation. The radiolabeling efficiency experiments were carried out to evaluate the complex yield of ^177^Lu‐DOTMP at regular time intervals by applying standard quality control techniques.

#### E.4 Biodistribution studies

Distribution of the radiolabeled complex was carried out in Wistar rats each weighing 200–250 g; two of the rats were sacrificed for each time point. Approximately 200μL of complex solution (pH=7) containing 5.5±0.05 MBq of ^177^Lu radioactivity was injected through the tail vein and the animals were sacrificed using CO_2_ asphyxiation at the end of 4 hrs, 1 day, 2 days, and 7 days postinjection. The tissues and organs were harvested, weighed, and rinsed with normal saline, and the activity associated with each organ was measured in a NaI (Tl) scintillation counter. Distribution of the activity in different organs was calculated as a percentage of injected activity (dose) per gram (%ID/g).

## III. RESULTS & DISCUSSION

### A. EXC separation

**Figure 1:**
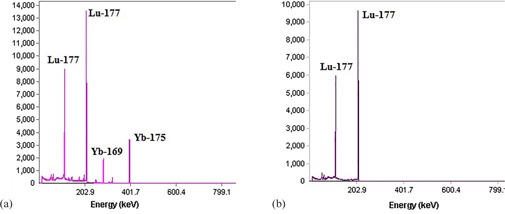
The gamma ray spectra of (a) the irradiated ^176^Yb (NO_3_)_3_ target and (b) the final product after the separation.

**Figure 2:**
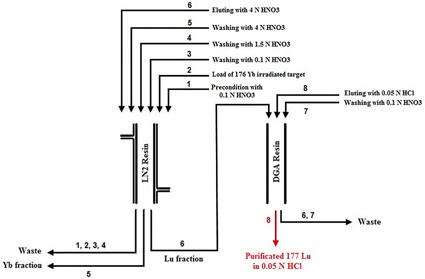
The flowsheet of EXC separation.

**Figure 3:**
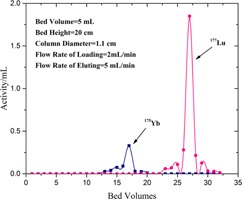
The resulting profile for the elution of ^177^Lu.

As previously was mentioned, many researchers investigated the separation macroquantities of ^177^Lu from Yb target. Balasubramanian^(36)^ described the production of NCA ^177^Lu by cation exchange chromatography using Dowex 50X8 (70% separation yield), Hashimato et al.^(37)^ reported the separation by reversed phase ion‐pair and (84% separation yield) in two works in 2003^(37)^ and 2015.^(40)^ Kumric et al.^(39)^ reported the separation using supported liquid membrane can separate ^177^Lu from Yb impurities. Also, Lahiri et al.^(38)^ extracted no‐carrier‐added ^177^Lu from proton activated Yb‐175 with HDEHP. The major disadvantage of above‐mentioned methods is the recovery of lanthanide from eluent (which needs further processing, it is time consuming, and suffers from loss of the ^177^Lu activity). Electrochemical separation^48^, ^49^ was applied to production of NCA ^177^Lu. Because of high cost of the enriched ^176^Yb, the recovery of target is very important. In this method, recovery of Yb target from mercury amalgam needs some chemical processing. In addition due to required material and equipment, this method is cost‐effective. A conceptual flowsheet was developed for the separation of ^177^Lu/^176^Yb by Horwitz et al.^(27)^ that is the base of separation in this work. EXC, as a separation strategy, is a combination of the liquid–liquid extraction and column chromatography; it also gains the selectivity and the rapidity of liquid–liquid extraction and column chromatography, respectively. In EXC separation, the irradiated target (the characteristics are shown in Table 1) was dissolved in dilute HNO_3_ (1 mL, 0.1 N). This solution containing ^175^Yb, ^169^Yb, and ^177^Lu was passed through the preconditioned column 1 (LN2 resin). The column was then washed with 30 mL of HNO_3_ 0.1 N and 1.5 N to remove ytterbium impurities. ^175^Yb radionuclide, as the major radionuclide impurity, was washed with HNO_3_ (50 mL, 4 N). The NCA ^177^Lu was eluted with HNO_3_ (50 mL, 4 N). In order to adjust the solution acidity and purification of ^177^Lu from the other metal ions, DGA resin was used in the next step. The collected solution of the previous step (^177^Lu in HNO_3_ (50 mL, 4 N)) was loaded onto the column 2 (DGA resin) and washed with HNO_3_ (30 mL, 0.1 N). The purified ^177^Lu was eluted with HCl (50 mL, 0.05 N). The gamma ray spectra of the irradiated target and the final product are shown in Fig. 1. No radiotracer of ytterbium radionuclide (^169^Yb, ^177^Yb, ^175^Yb) was observed in the γ spectrum of the ^177^Lu eluted portion. Various steps of radionuclides isolation are shown as a flowsheet in Fig. 2. Activity and the elution yield of each radionuclide in two separation steps on LN2 resin and DGA resin columns are given in Table 2. The elution's profile of ^177^Lu is shown in Fig. 3. The EXC has been considered as one of the potential procedure for the Lu/Yb separation due to the higher yield, relatively low concentration of acids, shortening time of the process, and minimizing the generation of wastes. As shown in Fig. 3, Yb and Lu are separated completely with no overlapping and broadening of the two peaks. Hence, for production of ^177^Lu, an enriched ytterbium target is so the economically target and the material recovery is another important aim in a selected separation procedure. Experimental data have shown that ytterbium could easily be extracted using an EXC column through washing the column followed by decreasing the acidity of solution without hard chemical processing. The overall recovery of NCA ^177^Lu was estimated as 82% and the overall processing time was as short as 3.5 hrs. To determine the optimum conditions, some effective factors were examined on separation Lu/Yb by EXC, including an initial mass of ytterbium target, flow rate of loading and elution, and the temperature.

**Table 1:** Characteristics of ytterbium isotope and radioisotopes from neutron reaction in reactor

*Isotope*	*Enriched (%)*	*(n*, γ*)*	*Half‐life*	*Cross‐section (barn)*	*Decay Mode*	*Decay Product*
^168^Yb	<0.12	^169^Yb	32.026 days	2300	EC	^169^Tm
^170^Yb	<0.12	^171^Yb	‐	9.9	stable	‐
^171^Yb	0.41	^172^Yb	‐	58.3	stable	‐
^172^Yb	0.69	^173^Yb	‐	1.3	stable	‐
^173^Yb	0.51	^174^Yb	‐	15.5	stable	‐
^174^Yb	1.8	^175^Yb	4.2 days	63	β‐,γ	^175^Lu
^176^Yb	96.4	^177^Yb	1.9h	2.85	β^‐^,γ	177Lu

**Table 2:** Activities and elution yield of separation processes on LN2 resin column 1 and DGA resin column 2

*Loading of 15.4 mCi* ^*177*^ *Lu and 2.7 mCi* ^*175*^ *Yb onto the Column 1 Containing LN2 Resin*				
	*Eluted Activity (mCi)*		*Eluted Yield (%)*	
*Separation Processes*	^177^Lu	^175^Yb	^177^Lu	^175^Yb
Washing the column 1 with HNO 0.1N	N.D^*^	N.D	‐	‐
Washing the column 1 with HNO 1.5N	N.D	4.07x10^‐4^	‐	0.015
Washing the column 1 with HNO 4N	6.32x10‐^3^	2.34	0.04	86
Elution the column 1 with HNO 4N	13.96	N.D	90	‐
Washing the column 2 with HNO 0.1N	3.41x10‐^5^	N.D	0.002	‐
Elution the column 2 with HCl 0.05 N	12.73	N.D	82	‐

#### A.1 Initial mass of ytterbium target

**Figure 4:**
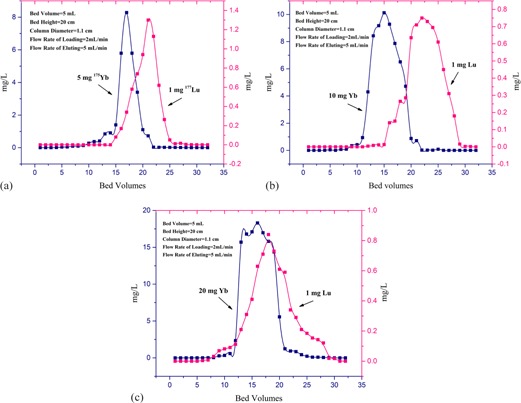
The effect of the initial mass of ytterbium (a) 5 mg, (b) 10 mg, and (c) 20 mg (bed volume = 5 mL, bed height = 20 cm, column diameter = 1.1 cm, flow rate of loading = 2 mL/min, and flow rate of eluting = 5 mL/min).

Figure 4 illustrates the effect of the initial mass of Yb on the resolutions of Lu and Yb. By increasing the amount of Yb from 5 to 20 mg a significant reduction in resolution occurred because of consuming a larger fraction of the column capacity and broadening of Lu peak considerably. So for separation in large quantities, using a column with the larger dimension and repeating the purification steps is necessary.

#### A.2 Temperature

**Figure 5:**
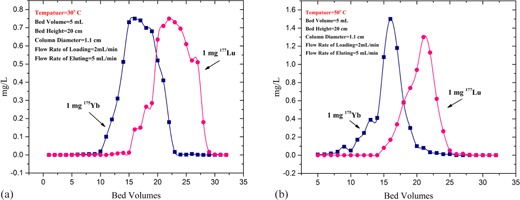
The effect of temperature on separation of Yb and Lu on column 1 (a) 30°C and (b) 50°C (Bed volume = 5 mL, bed height = 20 cm, column diameter = 1.1 cm, flow rate of loading = 2 mL/min, and flow rate of eluting = 5 mL/min).

Figure 5 shows the effect of temperature on separation of Yb and Lu on column 1 containing LN2 resin for 30°C and 50°C. Although the separation factor is higher at the lower temperature, the elution curves are broader, so the column 1 was thermostated at 50°C using recirculating water.

#### A.3 Flow rate of load and elution

Table 3 shows the effects of flow rate of load and elution on separation of Yb and Lu. A peristaltic pump was adjusted to obtain the optimized condition for loading of irradiated target on a column and eluting of NCA ^177^Lu.

**Table 3:** The effect of flow rate of load and elution

*Flow Rate of Loading (ml/min)*	*Flow Rate of Eluting (ml/min)*	*Time of Separation (hour)*	*Separation Yield (%)*
1	2	5	74
2	5	3.5	85
5	5	3	68.4
7	7	2.75	73

### B. Characterization of the radiolabeled ligands

**Figure 6:**
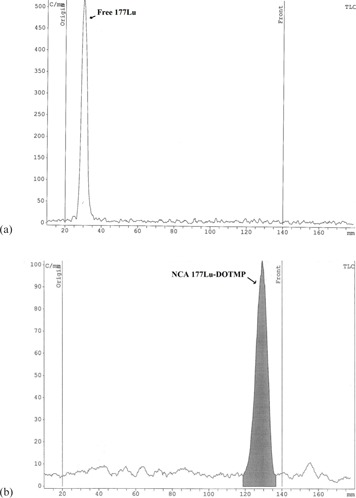
RTLC chromatographs for (a) the free NCA ^177^Lu and (b) the NCA ^177^Lu‐DOTMP in normal saline as eluent on Whatman paper.

The radiochemical yield was determined using RTLC. ^177^Lu‐DOTMP complex was characterized by employing paper chromatography technique using normal saline as the eluting solvent. It was observed that the complex moved towards the solvent front, while under identical conditions, the uncomplexed radiometal remained at the point of spotting (Fig. 6). NCA ^177^Lu–DOTMP complex was obtained in a very high yield (radiochemical purity > 98%) under the reaction conditions. The radiolabeling of DOTMP with ^177^Lu was reported by Chakraborty et al.^(6)^ and Das et al.^25^, ^51^ previously. ^177^Lu radionuclide was obtained by irradiation of natural lutetium (direct method). In this study ^177^Lu was obtained by irradiation of enriched ^176^Yb (indirect method). High specific activity is a significant characteristic of the NCA ^177^Lu that is produced by indirect method. Table 4 shows the specific activity of this work in comparison with other literature. No stable isotope carries the NCA ^177^Lu but, in direct method, product contains macroquantities of nonradioactive isotopes of Lu and, consequently, there will be a strong competition for the finite binding sites of the biolocalization agent between ^177^Lu and nonradioactive Lu cation. There is no significant difference between quality control activities in this study and the previously reported method. Reducing the amount of ligand used in formulation is still highly desirable. Therefore, one of the objectives was to reduce the amount of ligand; DOTMP was the sufficient amount of ligand in formulation to reach a high labeling yield complex formation (Table 5). NCA radionuclide with high specific activity and no isotope competition for binding need to minimum amount of ligand.

**Table 4:** The comparison of specific activity of ^177^Lu

	*Specific Activity (Ci/mg)*
This work	48
Das et al.^(51)^	0.324
Das et al.^(25)^	0.216
Chakraborty et al.^(6)^	0.324

**Table 5:** Effect of the amount of DOTMP on labeling efficiency

*DOTMP (mg)*	*Radiochemical Purity (%)*
0.2	96.1±0.3
0.6	98.2±0.1
1	98.7±0.15
3	99.1±0.13
6	99.3±0.1

### C. *In vitro* stability studies

The ^177^Lu‐DOTMP complex showed excellent stability when stored at pH ~ 7 at 37°C up to four half‐lives of the radionuclide; it was observed that the complex retains its radiochemical purity to the extent of > 95% after 30 days postpreparation. However, in similar work for carrier‐added (CA) ^177^Lu, radiochemical purity was decreased after .10 days postpreparation.

### D. Biodistribution

**Figure 7:**
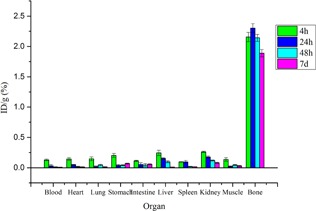
%ID/g of NCA ^177^Lu‐DOTMP in wild‐type rat tissues at 4 hrs, 24 hrs, 48 hrs, and 7 days postinjection.

The uptake of ^177^Lu–DOTMP complex in the different organs/tissue of Wistar rats, expressed as %ID per gram at different postinjection times, is shown in Fig. 7. The results of the biodistribution studies revealed the significant bone uptake (target tissue) within 4 hrs postinjection. ^177^Lu–DOTMP complex was rapidly taken up in the bone for 4 hrs after injection (ID/g%=2.15±0.07) and remained almost constant after seven days (ID/g%=1.9±0.06). Almost all the activity from blood was cleared into the bones within 4 hrs postinjection and no significant accumulation of activity was observed in any of the major organs/tissue at this time point. Lung, heart, intestine, stomach, and also muscle did not demonstrate significant uptake, except in kidneys and liver. However, the observed uptake in kidneys and liver were found to reduce with time; the activity injected was cleared via urinary excretion within 4 hrs postinjection. The measured uptake for bone in this study is also close to the 1.63 %ID/g measured by Das et al.^(51)^ The observed uptake in femur corresponding to a skeletal uptake of 36.11 %ID/organ for ^177^Lu‐DOTMP that is similar to the 36.58 %ID/organ measured by Chakraborty et al.^(6)^ As can be seen in this study and former works, ^177^Lu‐DOTMP showed higher uptake in bone and lower uptake in other major organs.

## IV. CONCLUSIONS

^177^Lu is a prospective reactor produced radionuclide and is suitable for palliation of bone pain. The results showed that under appropriate conditions and procedures, NCA ^177^Lu can be produced in a moderate flux reactor through irradiation of enriched ^176^Yb target and separation using the EXC procedure. The radionuclide purity of the ^177^Lu in final solution was obtained as 99.99%. Detecting the radiochemical yields by RTLC showed that the radiochemical purity of ^177^Lu‐DOTMP was higher than 98%. The biodistribution of the radiolabeled compound was checked in rat up to seven days, and rapid and selective skeletal uptake, fast clearance from blood, and almost no uptake in any of the major organs or tissue were observed. Therefore, the present study indicates that NCA ^177^Lu‐DOTMP has promising features and suggests good potentials for efficient use of this radio‐pharmaceutical to relief bone pain.

## COPYRIGHT

This work is licensed under a Creative Commons Attribution 3.0 Unported License.
